# Supplementary material for: Direct RNA sequencing on nanopore arrays redefines the transcriptional complexity of a viral pathogen
Source: Nat Commun. 2019 Feb 14;10:754. doi: 10.1038/s41467-019-08734-9 (PMC6376126; doi:10.1038/s41467-019-08734-9)
Supplement: Supplementary file 1 — Supplementary Information [file 41467_2019_8734_MOESM1_ESM.pdf]

Direct RNA sequencing on nanopore arrays redefines the transcriptional complexity of a viral pathogen

Supplementary Information

Depledge et al.

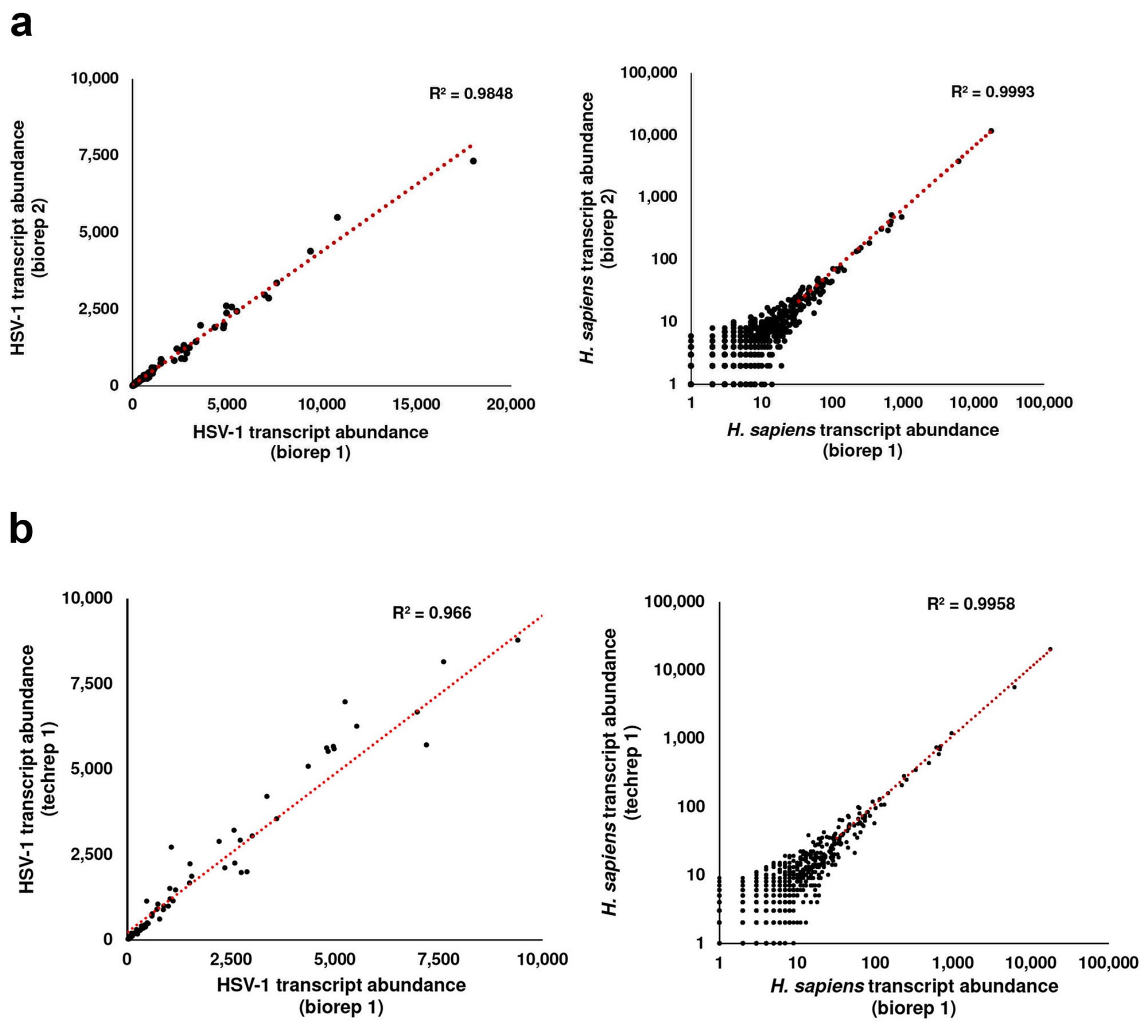

Supplementary Fig. 1: The reproducibility of direct RNA nanopore sequencing. **a,** transcript abundances were counted for the HSV-1 (left) and *H. sapiens* (right) transcriptomes and showed near perfect correlation between technical replicates of the NHDF 18hpi (i) and NHDF 18hpi (iii) samples, sequenced on separate MinION devices. **b,** transcript abundances were counted for the HSV-1 (left) and *H. sapiens* (right) transcriptomes and showed near perfect correlation between biological replicates of the NHDF 18hpi (i) and NHDF 18hpi (ii) samples. Here, each circle indicates the mRNA count for a discrete viral or host transcript.

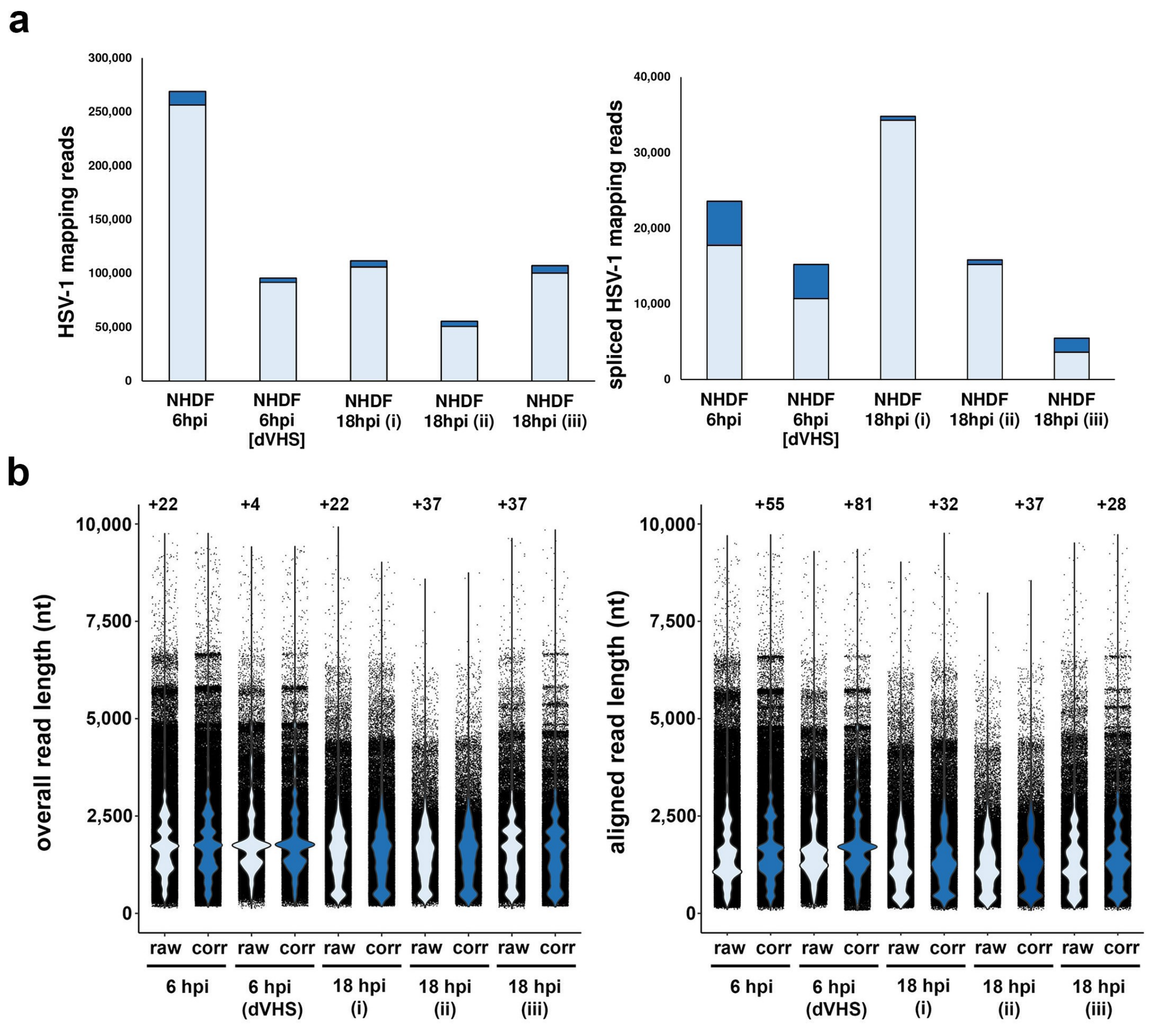

Supplementary Fig. 2: Error-correction notably improves nanopore sequence read alignment. a, error correction rescued a proportion of unmapped reads and reassigned these as HSV-1 mapping (left) and improved splice site detection in all datasets (right). Light blue bars indicate the HSV-1 mapping reads (left) and spliced HSV-1 reads (right) in the uncorrected dataset. Dark blue bars indicate the numbers of rescued reads following error correction. **b,** violin (dot) plots showing the distribution of overall sequence read lengths (left) and aligned read lengths (right) between raw and error-corrected reads. Overall sequence reads lengths are on average 4 - 37 nt longer in raw versus error-corrected datasets while the aligned read lengths are on average 28 – 81 nt longer in the error-corrected datasets.

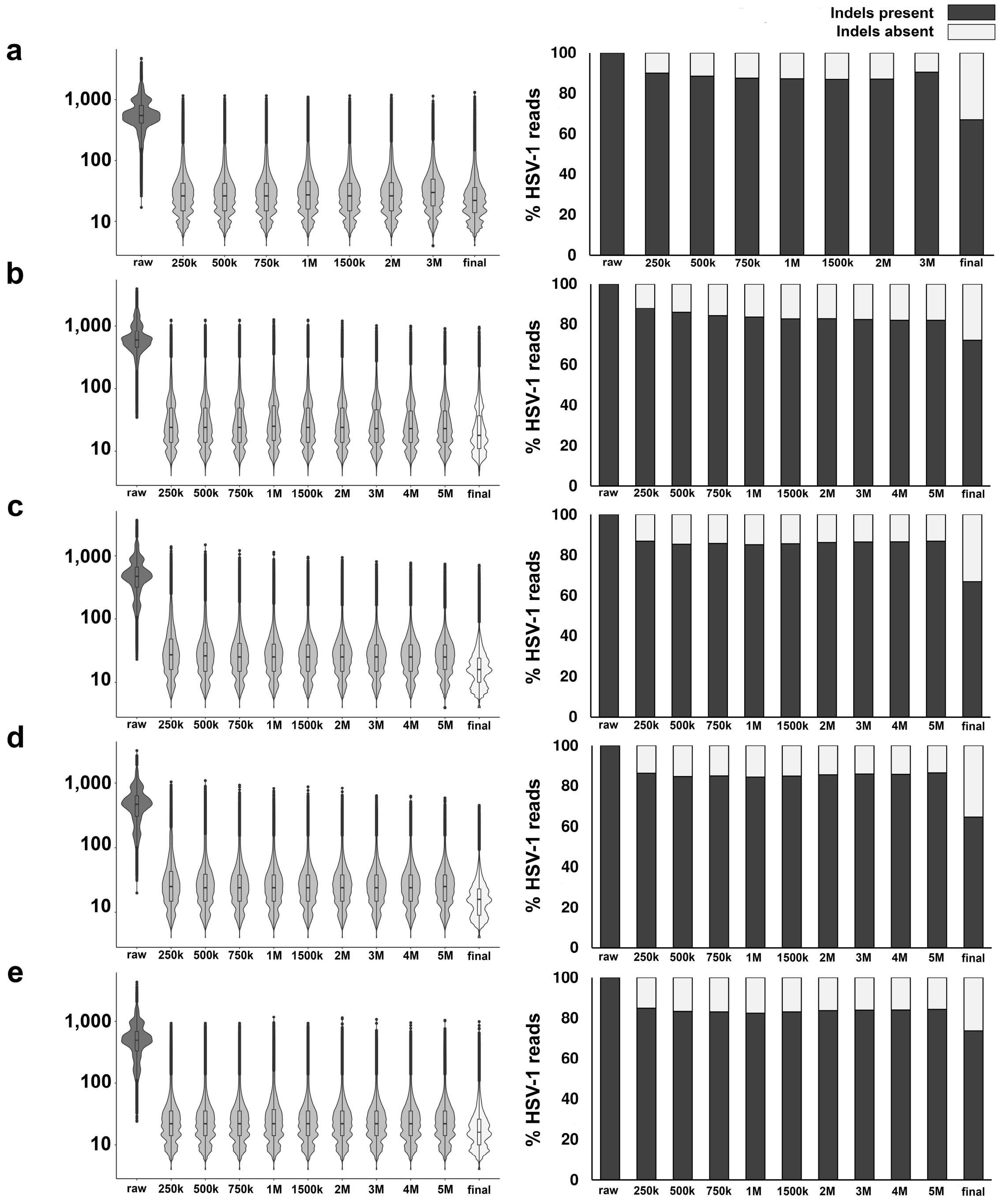

Indels present
Indels absent
a
b
c
d
e
% HSV-1 reads
raw 250k 500k 750k 1M 1500k 2M 3M 4M 5M final

Supplementary Fig. 3: Proovread error-correction significantly reduces the impact of indel and substitution type errors in nanopore direct RNA reads (HSV-1). For each of the five datasets generated in this study, sequence reads were mapped against the HSV-1 strain 17 genomes and the CIGAR string length (y-axis) extracted for each individual read. Here, violin plots (left) show the distributions of CIGAR string lengths and the effect of error-correction using proovread and FLASh-merged Illumina sequencing libraries, subsampled for varying numbers of reads (x-axis). Bar plots (right) denote the proportions of HSV-1 mapping reads that contain indels (denoted by the presence of I and/or D in CIGAR strings). Dark shading indicates the proportion of aligned reads containing indels post error-correction while light shading indicates the proportion with no indels following error-correction, The raw dataset comprises uncorrected nanopore reads while the final dataset contains the optimally corrected reads derived from a decision matrix (Fig. 2b), scored by shortest CIGAR string length. **a,** NHDF 6 hpi **b,** NHDF 6 hpi [HSV-1 dVHS mutant] (ii) **c,** NHDF 18hpi (i) biological replicate **d,** NHDF 18hpi (ii) biological replicate and **e,** NHDF 18hpi (iii) technical replicate on separate minION device.

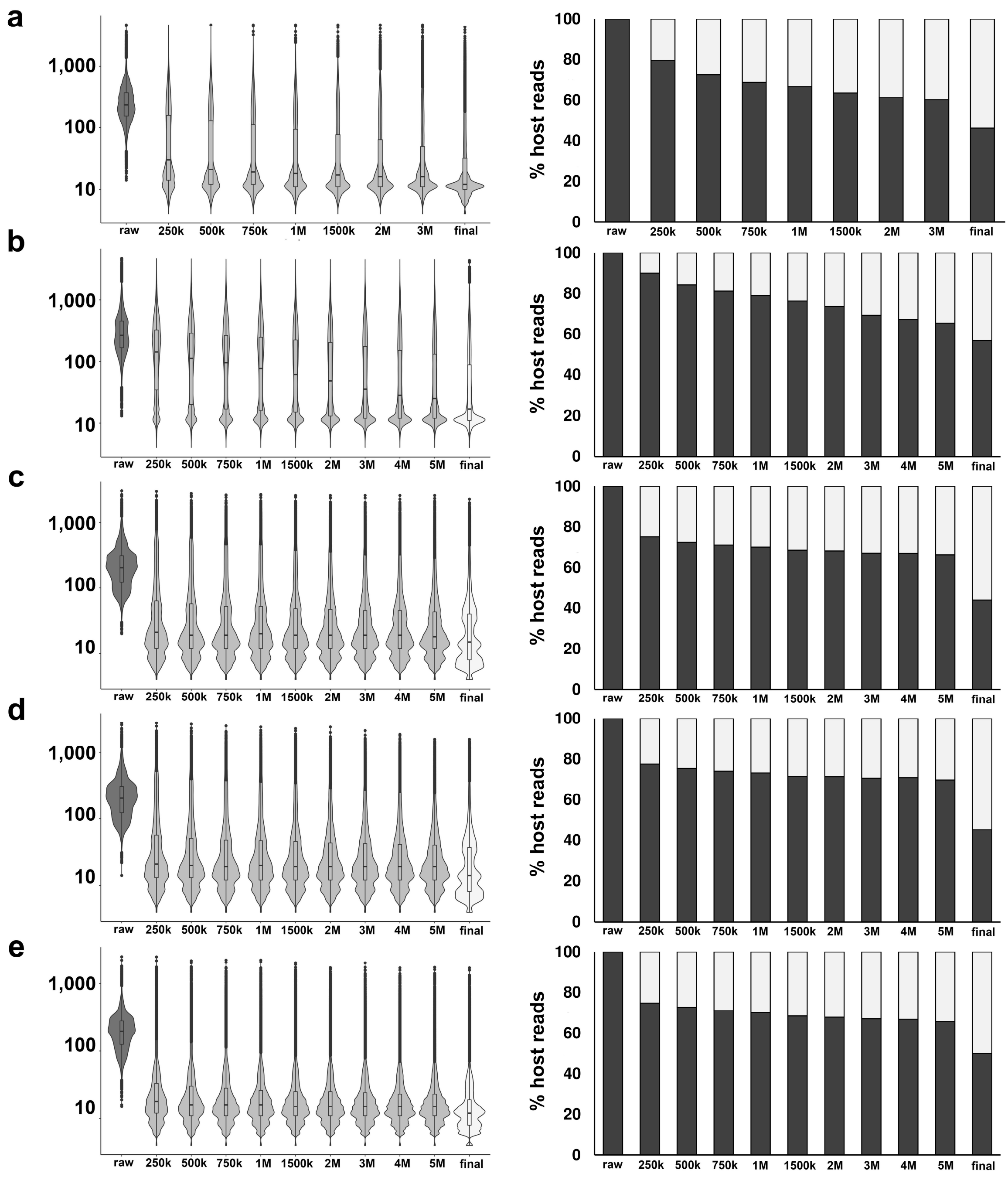
a
b
c
d
e
1,000
100
10
raw
250k
500k
750k
1M
1500k
2M
3M
4M
5M
final
% host reads
0
20
40
60
80
100

Supplementary Fig. 4: Proovread error-correction significantly reduces the impact of indel and substitution type errors in nanopore direct RNA reads (host). For each of the five datasets generated in this study, sequence reads were mapped against the *H. sapiens* transcriptomes and the CIGAR string length (y-axis) extracted for each individual read. Here, violin plots (left) show the distributions of CIGAR string lengths and the effect of error-correction using proovread and FLASh-merged Illumina sequencing libraries, subsampled for varying numbers of reads (x-axis). Bar plots (right) denote the proportions of *H. sapiens* mapping reads that contain indels (denoted by the presence of I and/or D in CIGAR strings). Dark shading indicates the proportion of aligned reads containing indels post error-correction while light shading indicates the proportion with no indels following error-correction, The raw dataset comprises uncorrected nanopore reads while the final dataset contains the optimally corrected reads derived from a decision matrix (Fig. 2b), scored by shortest CIGAR string length. **a,** NHDF 6 hpi **b,** NHDF 6 hpi [HSV-1 dVHS mutant] (ii) **c,** NHDF 18hpi (i) biological replicate **d,** NHDF 18hpi (ii) biological replicate and **e,** NHDF 18hpi (iii) technical replicate on separate minION device.

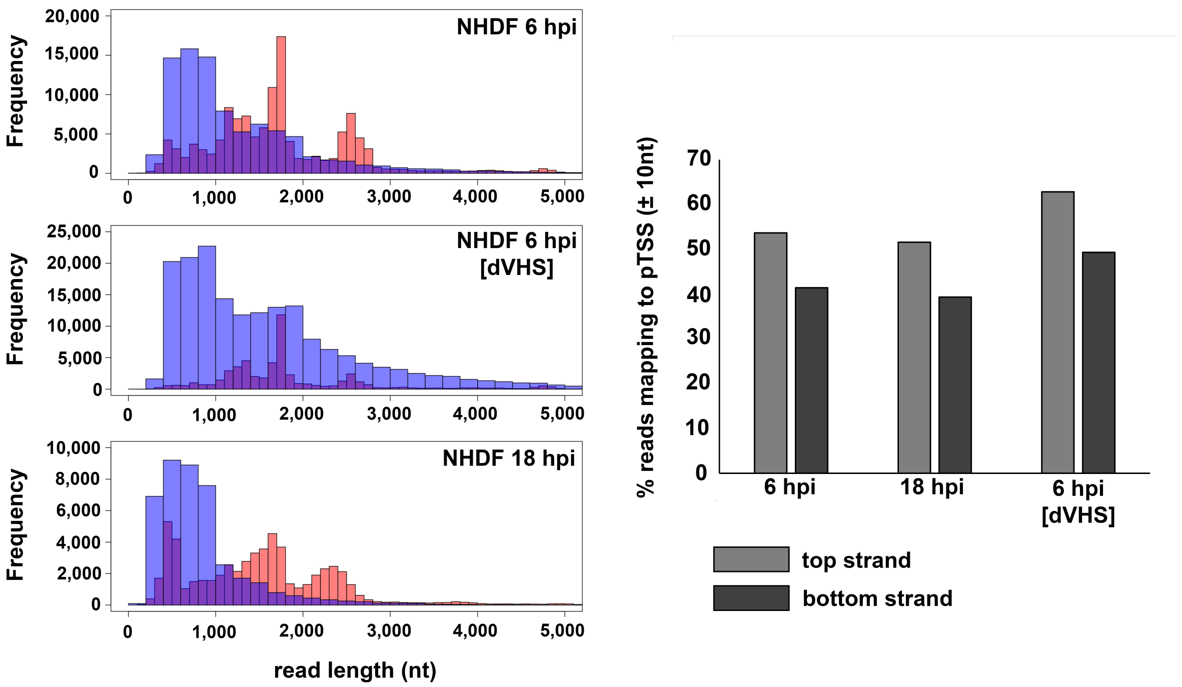

Supplementary Fig. 5: Host shutoff is impaired by an HSV-1 *vhs* mutant. a, read length distributions from NHDFs infected with wild type HSV-1 collected at 6 hpi (top) or 18 hpi (bottom), or infected with HSV-1 Δvhs collected at 6 hpi (middle). Read length distributions of human transcripts (blue) are extended at 6 h compared to 18 h. Viral reads are shown in red or purple. **b**, a greater fraction of reads from the top (shaded bar) or bottom (filled bar) strands of the HSV-1 genome have 5' ends that map close to the pTSS in cells infected with HSV-1 Δvhs compared to wild type.

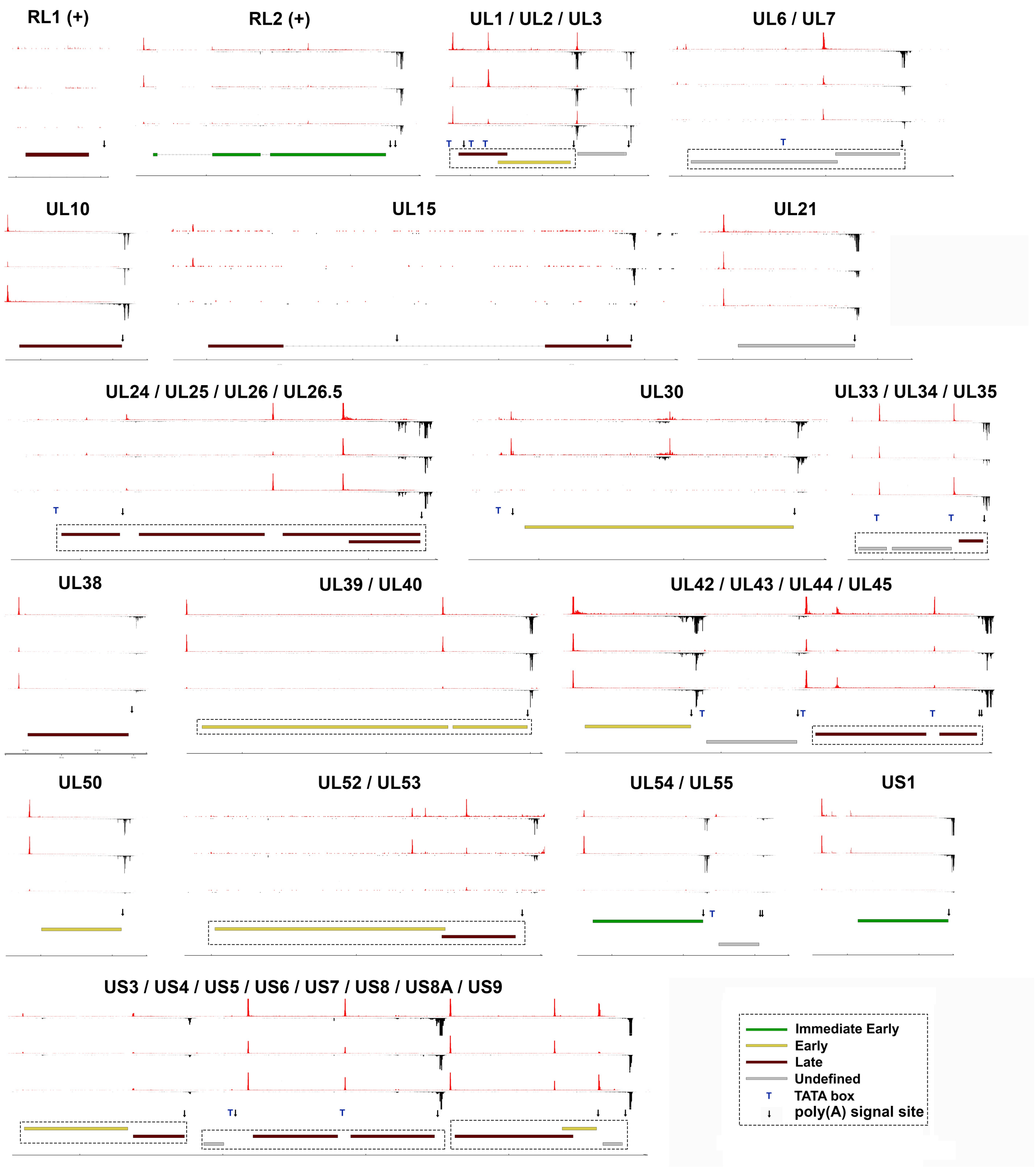
RL1 (+)
RL2 (+)
UL1 / UL2 / UL3
UL6 / UL7
UL10
UL15
UL21
UL24 / UL25 / UL26 / UL26.5
UL30
UL33 / UL34 / UL35
UL38
UL39 / UL40
UL42 / UL43 / UL44 / UL45
UL50
UL52 / UL53
UL54 / UL55
US1
US3 / US4 / US5 / US6 / US7 / US8 / US8A / US9
Immediate Early
Early
Late
Undefined
TATA box
poly(A) signal site

Supplementary Fig. 6: Identification of proximal transcription initiation and cleavage/polyadenylation sites along the top strand of the HSV-1 genome. Data sets correspond to NHDF infected with HSV-1 strain Patton for 6 hpi (upper track), strain F dVHS for 6 hpi (middle track) or strain Patton for 18 hpi (lower track). Transcripts are initiated a few nucleotides from the extreme 5' end of each nanopore read (red peaks) and polyadenylation (black peaks) occurs downstream of canonical (AAUAAA) PAS sequences. Canonical HSV-1 ORFs are colored according to kinetic class (IE – green, E – yellow, L – red, undefined – grey) while polycistronic transcriptional units are indicated by hatched boxes. Identifiable TATA boxes are indicated by an inverted blue T. Transcription proceeds from left to right. Note only the regions encompassing canonical ORFs are shown.

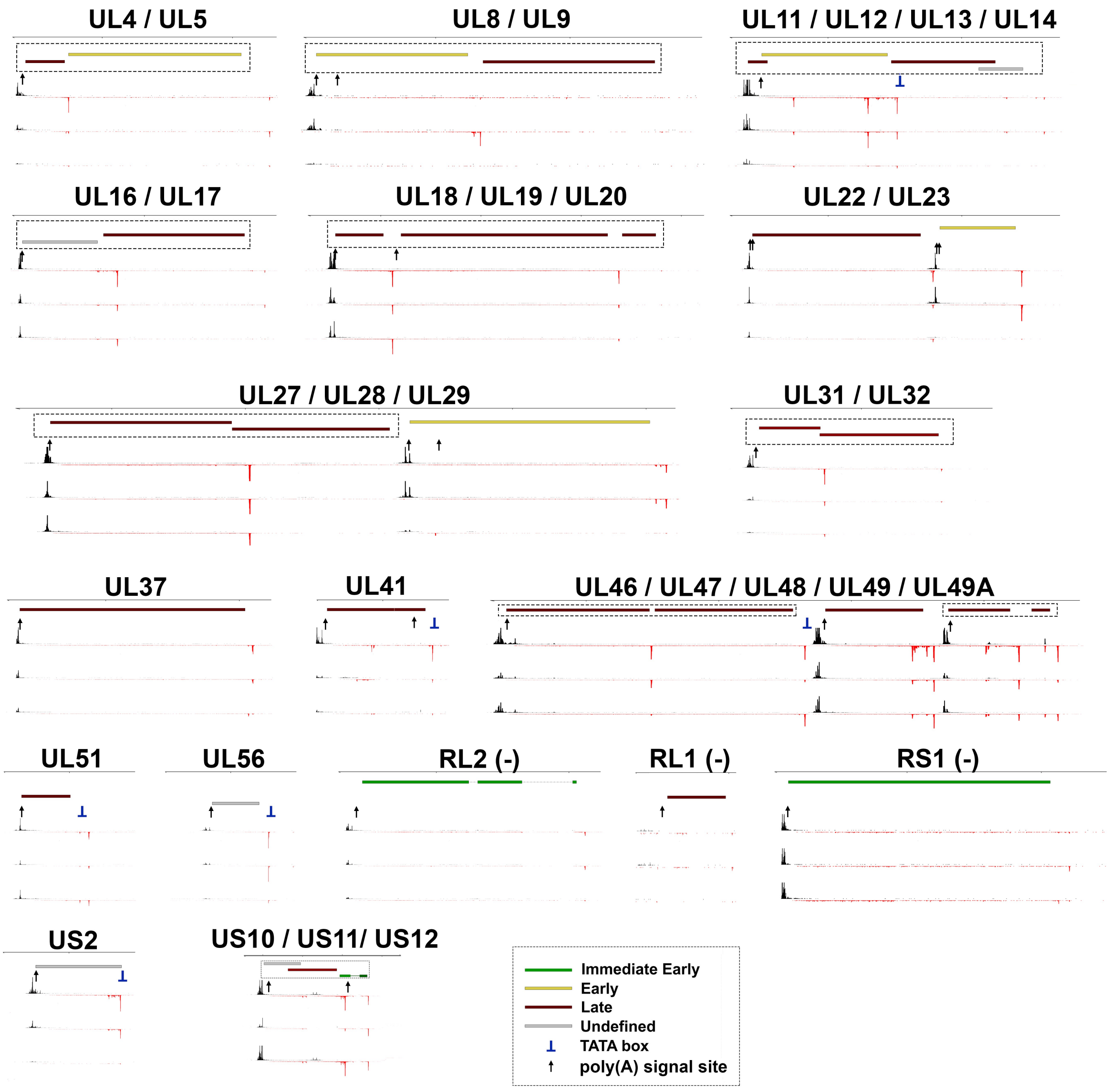

Supplementary Fig. 7: Identification of proximal transcription initiation and cleavage/polyadenylation sites along the bottom strand of the HSV-1 genome. Data sets correspond to NHDF infected with HSV-1 strain Patton for 6 hpi (upper track), strain F dVHS for 6 hpi (middle track) or strain Patton for 18 hpi (lower track). Transcripts are initiated a few nucleotides from the extreme 5' end of each nanopore read (red peaks) and polyadenylation (black peaks) occurs downstream of canonical (AAUAAA) PAS sequences. Canonical HSV-1 ORFs are colored according to kinetic class (IE – green, E – yellow, L – red, undefined – grey) while polycistronic transcriptional units are indicated by hatched boxes. Identifiable TATA boxes are indicated by an inverted blue T. Transcription proceeds from right to left. Note only the regions encompassing canonical ORFs are shown.

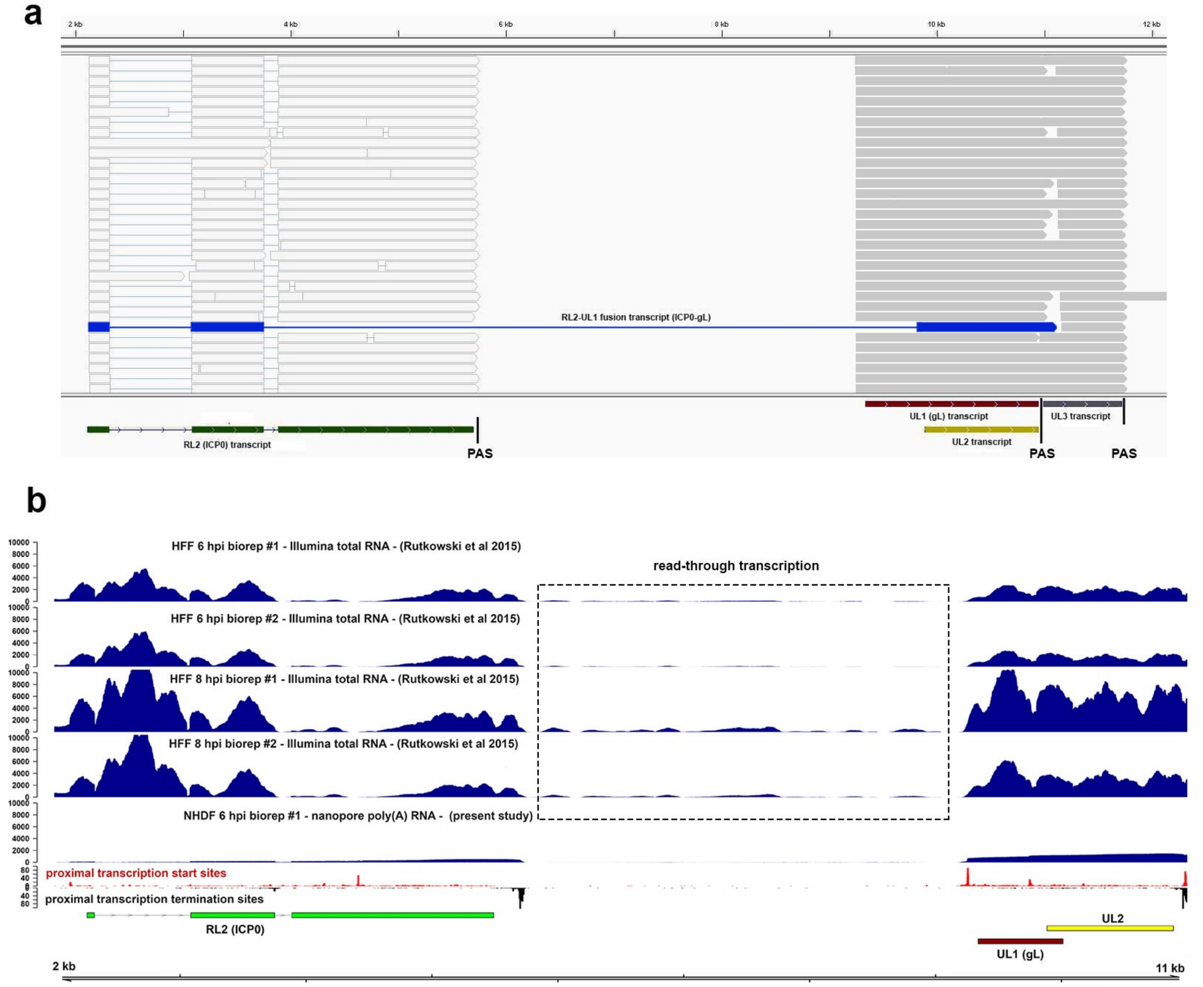

Supplementary Fig. 8: IGV screenshots illustrating read-through transcription across the RL2-UL1 locus. a, Integrative Genomics Viewer (IGV) close up of the 5' end of the unique long region of the HSV-1 genome orientated so that transcription is from left to right. Canonical transcripts are labelled and colored according to kinetic class (IE - green, E - yellow, L - red, undefined - grey). Nanopore sequence reads are shown as either white (multi-mapping –two copies of RL2 are present in the genome) or grey (uniquely-mapping). An example of the RL2-UL1 fusion transcript that encodes ICP0-gL is highlighted in blue. **b,** Illumina datasets (top four tracks) derived from sequencing of ribosomal RNA-depleted total RNA were downloaded from the Rutkowski et al 2015 study and reanalyzed to demonstrate the presence of low-level read-through transcription across the RL2-UL1 locus. Direct RNA sequencing generated from this study is also included (track 5) as are the proximal transcription start sites and transcription termination sites (RNA cleavage sites). The genomic region showing evidence of read-through transcription is indicated by the hatched box.

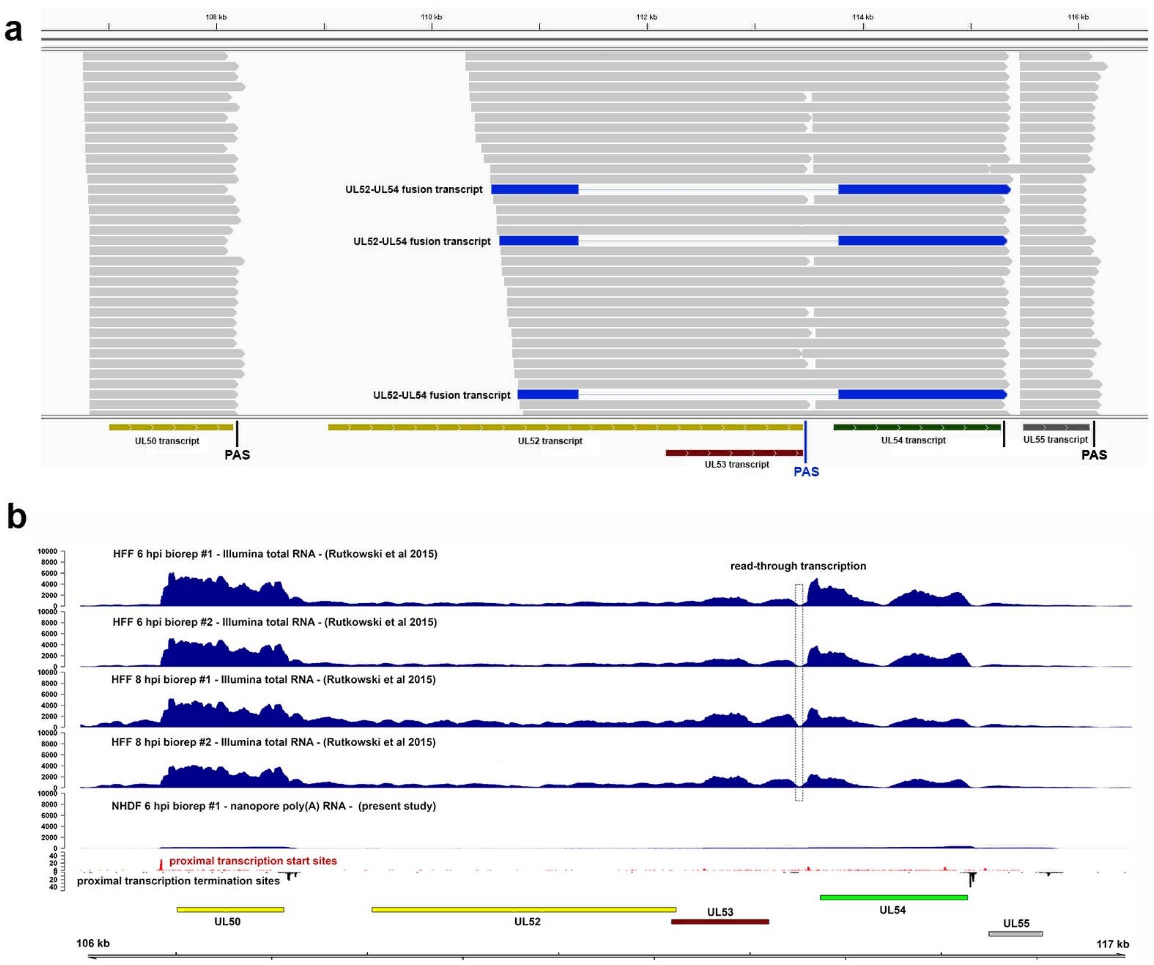

Supplementary Fig. 9: IGV screenshots illustrating read-through transcription across the UL52-UL54 locus. a, Integrative Genomics Viewer (IGV) close up of the 3' end of the unique long region. Transcripts are labelled and orientated as in (a). Note that polyadenylated RNAs are read 3'->5', and as such the 5' mapping of sequence reads may be influenced by RNA decay. Transcripts giving rise to the putative UL52-UL54 fusion are highlighted in blue. Canonical PAS sites (AAUAAA) are shown as vertical black bars while the non-canonical PAS sites (AUUAAA) is shown as a vertical blue bar. **b,** Illumina datasets (top four tracks) derived from sequencing of ribosomal RNA-depleted total RNA were downloaded from the Rutkowski et al 2015 study and reanalyzed to demonstrate the presence of low-level read-through transcription across the UL52-UL54 locus. Direct RNA sequencing generated from this study is also included (track 5) as are the proximal transcription start sites and transcription termination sites (RNA cleavage sites). The genomic region showing evidence of read-through transcription is indicated by the hatched box.

Run name	device	Run type	Total input RNA (µg)	Total Reads	Pass reads	Fail reads	CS reads
NDHF 6hpi	MinION Mk Ib [Unit B]	Direct RNA (minION)	25	519,417	373,625	124,826	20,966
NDHF 6hpi [dVHS]	MinION Mk Ib [Unit B]		21.2	422,274	300,516	114,080	7,678
NHDF 18hpi (i)	MinION Mk Ib [Unit A]		25	377,163	218,903	114,374	43,886
NHDF 18hpi (ii)	MinION Mk Ib [Unit A]		25	217,022	112,700	90,912	13,410
NHDF 18hpi (iii)	MinION Mk Ib [Unit B]		25	297,866	169,515	64,299	64,052
NHDF 18hpi (iii)	Illumina HiSeq 4000	Illumina mRNA-Seq	2	24,929,213	n/a	n/a	n/a

all native RNA sequencing runs were performed for 18 hours

CS – RNA calibration strand reads

n.a. – not applicable

Supplementary Table 1: Sequencing metrics

Transcription unit (first encoded ORF)	5' transcript end peak* (proximal transcript start)	Coding strand	nearest TATAAA box (upstream)	Distance to upstream TATA box (nt)	Distance to ATG in first encoded ORF
RL1 (+)	425	+	n/a	n/a	n/a
RL2 (+)	2129	+	n/a	n/a	133
UL1 / UL2	9257	+	9217	-40	81
UL2	9749	+	9713	-36	136
UL3	10984	+	n/a	n/a	7
UL6 / UL7	15058	+	n/a	n/a	73
UL7	16963	+	n/a	n/a	173
UL10	23041	+	n/a	n/a	164
UL15	28812	+	n/a	n/a	209
UL21	41872	+	n/a	n/a	203
UL24 / UL25 / UL26 / UL26.5	47418	+	n/a	n/a	320
UL25 / UL26 / UL26.5	48641	+	n/a	n/a	173
UL26 / UL26.5	50675	+	n/a	n/a	135
UL26.5	51646	+	n/a	n/a	82
UL30	62618	+	n/a	n/a	189
UL33 / UL34 / UL35	69075	+	n/a	n/a	86
UL34 / UL35	69453	+	69412	-41	180
UL35	70497	+	70456	-41	69
UL38	84404	+	n/a	n/a	127
UL39	86227	+	n/a	n/a	206
UL40	89786	+	n/a	n/a	139
UL42	92945	+	n/a	n/a	167
UL43	94744	+	n/a	n/a	55
UL44 / UL45	96171	+	96141	-30	141
UL45	97963	+	n/a	n/a	70
UL50	106844	+	n/a	n/a	167
UL52 / UL53	n.d.	+	n/a	n/a	n/a

UL53	111953	+	n/a	n/a	227
UL54	113613	+	n/a	n/a	122
UL55	115458	+	115416	-42	39
LAT (+)	n.d.	+	n/a	n/a	n/a
US1	132142	+	n/a	n/a	505
US3 / US4	135204	+	n/a	n/a	21
US4	136754	+	n/a	n/a	-7
US5 / US6 / US7	137638	+	n/a	n/a	96
US6 / US7	138358	+	n/a	n/a	65
US7	139712	+	139677	-35	77
US8 / US8A / US9	141183	+	n/a	n/a	64
US8A / US9	142641	+	n/a	n/a	107
US9	143260	+	n/a	n/a	57
RS1 (+)	146938	+	n/a	n/a	128
LAT (-)	n.d.	-	n/a	n/a	n/a
UL5 / UL4	12488	-	n/a	n/a	65
UL5	15572	-	n/a	n/a	440
UL9 / UL8	20666	-	n/a	n/a	95
UL9	n.d.	-	n/a	n/a	n/a
UL11	25488	-	n/a	n/a	396
UL12 / UL11	27035	-	27076	-41	147
UL13 / UL12 / UL11	28676	-	n/a	n/a	173
UL14 / UL13 / UL12 / UL11	29236	-	n/a	n/a	320
UL16 / UL17	31597	-	n/a	n/a	301
UL17	33802	-	n/a	n/a	304
UL18 / UL19 / UL20	36239	-	n/a	n/a	187
UL19 / UL20	40756	-	n/a	n/a	227
UL20	41603	-	n/a	n/a	114
UL22	46570	-	n/a	n/a	187

UL23	47897	-	n/a	n/a	94
UL28 / UL27	56070	-	n/a	n/a	275
UL28	58848	-	n/a	n/a	688
UL29	62220	-	n/a	n/a	249
UL32 / UL31	67448	-	n/a	n/a	69
UL32	69208	-	n/a	n/a	16
UL36	n.d.	-	n/a	n/a	n/a
UL37	84136	-	n/a	n/a	52
UL41	92743	-	92785	-42	107
UL47 / UL46	100982	-	n/a	n/a	29
UL47	103300	-	103341	-41	183
UL48	105247	-	n/a	n/a	167
UL49A / UL49	106528	-	n/a	n/a	136
UL49A	107117	-	n/a	n/a	123
UL51	109157	-	109196	-39	145
UL56	117068	-	117116	-48	142
RL2 (-)	124246	-	n/a	n/a	134
RL1 (-)	n.d.	-	n/a	n/a	n/a
RS1 (-)	n.d.	-	n/a	n/a	n/a
US2	135293	-	135336	-43	362
US12 / US11 / US10	145160	-	n/a	n/a	60
US12 / US11	145452	-	n/a	n/a	201
US12	146054	-	n/a	n/a	472

Supplementary Table 2: pTSS for canonical HSV-1 ORFs

Transcription unit (first encoded ORF)	5' transcript end peak* (proximal transcript start)	Coding strand	nearest TATAAA box (upstream)	Distance to upstream TATA box (nt)	Distance to ATG in first encoded ORF	Note
UL6 / UL7	14938	+	n/a	n/a	193	
	15026	+	n/a	n/a	105	
	15058	+	n/a	n/a	73	largest peak
UL24 / UL25 / UL26 / UL26.5	47418	+	n/a	n/a	320	largest peak
	47681	+	n/a	n/a	57	
UL53	111771	+	n/a	n/a	409	
	111953	+	n/a	n/a	227	largest peak
US1	132142	+	n/a	n/a	505	largest peak
	132542	+	n/a	n/a	105	
UL9 / UL8	20572	-	n/a	n/a	95	largest peak
	20666	-	n/a	n/a	189	
UL14 / UL13 / UL12 / UL11	29236	-	n/a	n/a	320	largest peak
	29115	-	n/a	n/a	199	
UL29	62303	-	n/a	n/a	249	
	62220	-	n/a	n/a	166	largest peak
	62151	-	n/a	n/a	97	
UL51	109157	-	109196	-39	145	
	109293	-	n/a	n/a	181	largest peak

Supplementary Table 3: ORFs with multiple pTSS

5' transcript end peak* [proximal transcript start]	Transcription unit	Coding strand	Description
15860	UL6/UL7	+	internal to UL6 ORF
48087	UL24/UL25/UL26/UL26.5	+	internal to UL24 ORF
64808	UL30	+	internal to UL30 ORF
96607	UL44/UL45	+	internal to UL44 ORF | Previously described by Tombácz et al 2017
111953	UL52/UL53	+	internal to UL53 ORF
115408	UL55	+	internal to UL55 ORF | Previously described by Tombácz et al 2017
26748	UL11/UL12/UL13/UL14	-	internal to UL12 ORF | Previously described by Tombácz et al 2017
91834	UL41	-	internal to UL41 ORF | Previously described by Tombácz et al 2017

Supplementary Table 4: Novel pTSS

<table>
<tr><th>Fusion</th><th>exons</th><th>Exon 1 start</th><th>Exon 1 end</th><th>Exon 2 start</th><th>Exon 2 end</th><th>Exon 3 start</th><th>Exon 3 end</th></tr>
<tr><td rowspan="3">RL2 - UL1</td><td rowspan="3">3</td><td rowspan="3">2261</td><td>2318</td><td>3083</td><td>3750</td><td>9821</td><td rowspan="3">10012</td></tr>
<tr><td>GAG|GTGAGG</td><td>TTAG|C</td><td>ACG|GTGAGG</td><td>GTAG|G</td></tr>
<tr><td>fully conserved</td><td>fully conserved</td><td>fully conserved</td><td>fully conserved</td></tr>
<tr><td rowspan="3">UL52 - UL54</td><td rowspan="3">2</td><td rowspan="3">109049</td><td>111360</td><td>113787</td><td rowspan="3">115273</td><td></td><td></td></tr>
<tr><td>CCC|GTACGT</td><td>ACAG|C</td><td></td><td></td></tr>
<tr><td>fully conserved</td><td>fully conserved</td><td></td><td></td></tr>
</table>

intron sequence in splice donor and acceptor shown in italics

all genome co-ordinates refer to HSV-1 strain 17 (NC_001806.2)

Supplementary Table 5: Fusion transcripts

Target	Start	Stop	Sequence
RL2 exon2 – exon3 splice junction	3720	3737	ACGGACGAGGATGACGAC
	4066	4047	TGGTGGTGGTGTTGGTGTTA
RL2 exon2 – UL1 internal splice junction	3615	3634	GCCGTGGACTTTATCTGGAC
	9857	9838	CAGACGTTCCGTTGGTAGGT
UL1 internal	9734	9751	CTGGACAGTCGCAAGCAG
	9838	9857	CAGACGTTCCGTTGGTAGGT
UL52 internal	111235	111254	CGAGGTTGCCTACTTTGACC
	111366	111385	CGTTAGAGAACCGTGGACGA
UL54 internal	113737	113756	GGCGACTGACATTGATATGC
	113874	113892	GGGTCTTCCATGTCCTCGT
UL52 - UL54 internal splice junction	111235	111254	CGAGGTTGCCTACTTTGACC
	113874	113892	GGGTCTTCCATGTCCTCGT
18s rRNA	Chr 21		AGGAATTGACGGAAGGGCAC
	Chr 21		TTATCGGAATTAACCAGACA

Note: all genome co-ordinates refer to HSV-1 strain 17 (NC_001806.2)

Supplementary Table 6: Primers used in this study

Supplementary Note 1

During the course of this study, we observed unexpected artefacts in a small number of nanopore sequence reads. These became apparent when profiling the CIGAR strings for each aligned read to identify spliced transcripts. We exemplify this here by focusing on the sequencing of the RNA CS (Enolase 2) control strand. Enolase 2 is a single exon gene that is not known to undergo splicing (https://goo.gl/TepUW5).

Here, we align sequence reads passing QC using MiniMap2 and determine the number of mapping reads and the number of reads containing 'N' characters in their CIGAR string. 'N' is defined as a skipped region from the reference and denotes the presence of a presumed intron.

```
#map reads
minimap2 -ax splice -k14 -uf --secondary=no ENO2_RNACS.fasta QCpass.reads.fastq >
outfile.cs.sam
#determine total reads mapped (flag 0)
cut -f2 outfile.cs.sam | sort | uniq -c
#determine number of reads with 'internal splicing'
awk '$6 ~/N/ {print $0}' outfile.cs.sam | wc -l
```

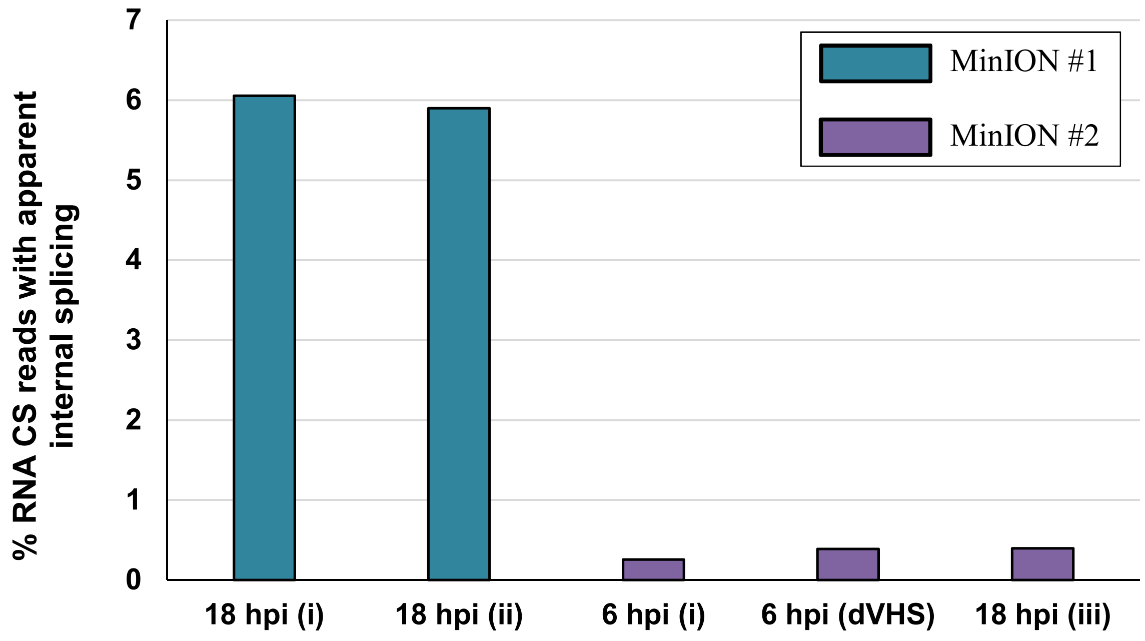

The reader will note that the % of RNA CS mapping reads is heavily influenced by the specific MinION device used (each was plugged into a different computer in a different building). We repeated basecalling using several previous version of the albacore basecaller

without any notable changes. We discussed our data with customer support at Oxford Nanopore Technologies but remain unable to discern why this occurs.

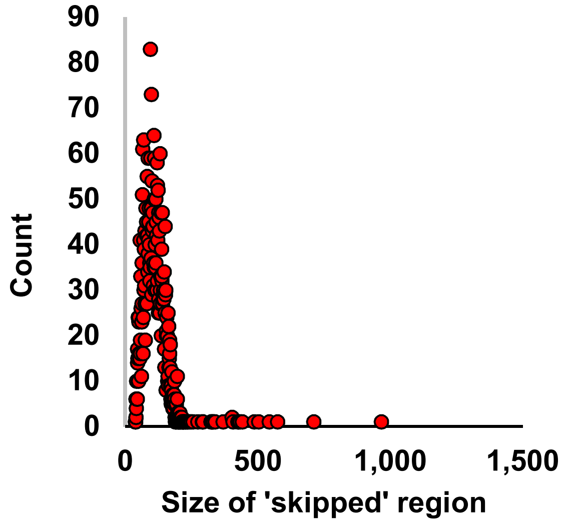

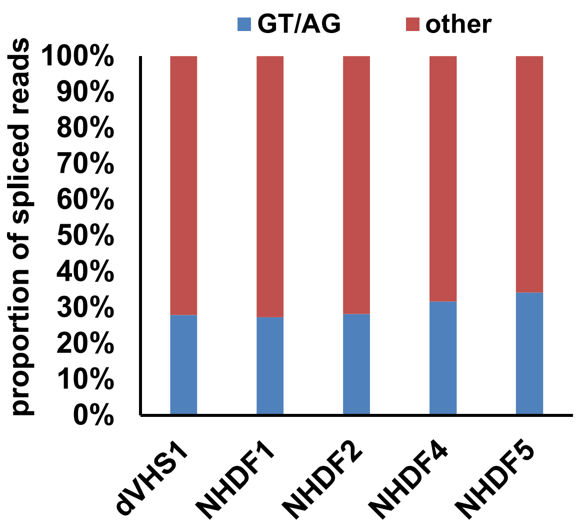

Figure 2: (left) The sizes of the 'intron' regions within spliced reads were determined and collated for all five datasets. The median size of the 'introns' is 95nt. (right) only 25-30% of the 'splice junctions' utilize canonical GT/AG splice donor/acceptor pairs.

The positions and sizes of the artefactual introns are inconsistent, suggesting it is not a sequence feature. Indeed, to most notable features are that (i) the intron lengths are generally < 100 nt, and (ii) multiple reads containing the same intron artefact are not seen. Splice donor/acceptor sequences tend toward canonical GT/AG pairs but this is likely a manifestation of MiniMap2 attempting to align in a splice-aware manner.

From the point of view of our studies, the impact was minimal as we were generally able to distinguish artefactual introns from real introns by examining splice site usage and, most critically, requiring that identical introns are identified in multiple distinct sequence

reads. Finally, of course, experimental validation was used to confirm the novel splice junctions we discovered.

Supplementary Note 2:
Examples of code used during data processing and analysis

Introduction

-- The aim of this document is to provide guidance on how to perform the same analyses as those presented in this manuscript. We can offer no guarantees as to how translatable these approaches are to other studies but hope that the following will be of use to those interested in replicating or performing similar studies. Readers should also note that much of the simple data processing was performed using simple command line tools (i.e. awk and sed) while other steps were processed manually using spreadsheet software. A full list of softwares required to run these analyses are detailed at the end of this document. Please note that we are not responsible for these softwares and as many are continually updated, certain commands or functions may change or become redundant. Please remember that questions about errors when installing/using softwares listed here should be directed at the authors of the software and not the authors of this manuscript. Specific questions regarding any of the below may be directed to the corresponding authors listed in the manuscript.

Error-correction

-- Our error-correction approach requires a compatible Illumina dataset, ideally generated from the same source material. Importantly, error-correction works best with longer Illumina paired-end reads (e.g. 2 x 100) that are merged using FLASh. However, simply extending the number of sequencing cycles used is insufficient as fragment lengths should also be increased. Most Illumina poly(A) selection protocols perform a fragmentation step at 96-98^{0}C for 8-10 mins. By reducing temperature (e.g. 92^{0}C) and time (e.g. 6 mins), the median size of RNA fragments can be pushed toward 250 – 300 nt). Following FLASh merging of the Illumina dataset, nanopore reads are separated into groups (Chunks) and corrected using Illumina data.

```
#1: Generate FLASh merged Illumina dataset
FLASh --min-overlap=10 --max-overlap=150 illumina_1.fq illumina_2.fq
#2: Convert raw nanopore sequence reads (fastq) to fasta form with T replacing U
fastq2fasta -i raw.fastq -o raw.fasta
sed 's/U/T/g' raw.fasta > raw_U2T.fasta
#3: Use SeqChunker (proovread package) to chunk reads* and run proovread** using
subsampled Illumina reads to perform error-correction
/proovread/bin/SeqChunker -s 4M -o SeqChunk.%03d.fa raw_U2T.fasta
```

```
/proovread/bin/proovread -l SeqChunk.001.fa -s extendedFrags.fastq -s notCombined_1.fastq -s notCombined_2.fastq --pre proovread-001 --overwrite --no-sampling
```

*chunking sizes can be adjusted (e.g 8M, 16M) if the number of individual SeqChunks becomes too high to manage

-- The proovread step should be run for every SeqChunk generated and for every subsampled Illumina dataset. This is very intensive and should be parallelised as much as possible using an HPC cluster.

-- The final output from each proovread run should be a directory containing a file ending in *untrimmed.fq. The untrimmed.fq files generated from each SeqChunk should be concatenated into a single fastq file that subsequently contains all error-corrected reads resulting from a specific subsampled Illumina dataset. These can then be mapped against the genome of choice and processed to extract CIGAR string lengths

Determining the overall lengths of HSV-1 mapping pseudotranscripts

#1: align raw and error-corrected fastq datasets to the HSV-1 reference genome

```
minimap2 -ax splice -k14 -uf --secondary=no refgenome.fasta reads.fq > reads.outfile.sam
```

#2: filter for primary mapping reads only (flags 0 & 16) and use egrep and AWK to determine overall read length of each individual sequence read and print to stdout

```
egrep HSV1-st17 reads.outfile.sam | grep -v 2048 | grep -v 2064 | cut -f10 | awk '{ print length($0); }'
```

---For visualization options, see section 'Visualization using Rstudio'

Determining the aligned lengths of HSV-1 mapping pseudotranscripts

#1: align raw and error-corrected fastq datasets to the HSV-1 reference genome

```
minimap2 -ax splice -k14 -uf --secondary=no refgenome.fasta reads.fq > reads.outfile.sam
```

#2: use bamutils (ngsutils) to trim soft-clipped sequence reads (i.e. remove the soft-clipped part of the sequence)

```
samtools view -o reads.outfile.bam reads.outfile.sam
bamutils removeclipping reads.outfile.bam reads.outfile.clipped.bam
samtools view reads.outfile.clipped.bam > reads.outfile.clipped.sam
```

#3: filter for primary mapping reads only (flags 0 & 16) and use egrep and AWK to determine overall read length of each individual sequence read and print to stdout

```
egrep HSV1-st17 reads.outfile.clipped.sam | grep -v 2048 | grep -v 2064 | cut -f10 | awk '{ print length($0); }'
```

---For visualization options, see section 'Visualization using Rstudio'

Determine proportion of reads encoding ORFs > 90 nt

#1: align pseudotranscripts to HSV-1, convert pseudotranscripts from fastq to fasta

```
minimap2 -ax splice -k14 -uf --secondary=no refgenome.fasta pseudotranscripts.fq > outfile.sam
fastq2fasta -i pseudotranscripts.fq -o pseudotranscripts.fasta
```

#2: extract mapping read IDs and deduplicate, then use BBtools (filterbyname.sh) and ORF identification script (https://goo.gl/bgbMEs)

#top strand

```
cut -f1,2 outfile.sam | grep -w 0 | cut -f1 | sort | uniq > ids.list
filterbyname.sh in= pseudotranscripts.fasta out=pseudo.mapped.fasta names=ids.list include=t
```

```
python ~/scripts/get_orfs_or_cdss.py -i pseudo.mapped.fasta -f fasta -t CDS -e closed -m one -s forward --min_len=90 --on longestORFs.forward.fasta
#bottom strand
cut -f1,2 outfile.sam | grep -w 16 | cut -f1 | sort | uniq > ids.list
filterbyname.sh in= pseudotranscripts.fasta out= pseudo.mapped.fasta names=ids.list include=t overwrite=true
python ~/scripts/get_orfs_or_cdss.py -i pseudo.mapped.fasta -f fasta -t CDS -e closed -m one -s reverse --min_len=90 --on reverse.longestORFs.fasta
#3: determine number of ORFs present
cat longestORFs.forward.fasta reverse.longestORFs.fasta > longestORFs.fasta
grep -c \> longestORFs.fasta
```

Mapping 5' pTSS and 3' pTTS sites

```
#1: Map reads to HSV-1 genome and parse to sorted BAM file (requires SAMtools and BEDtools)
minimap2 -ax splice -k14 -uf --secondary=no refgenome.fasta reads.fq > reads.outfile.sam
samtools view -b -F4 -o reads.outfile.bam reads.outfile.sam
samtools sort -o reads.outfile.sorted.bam reads.outfile.bam
samtools index reads.outfile.sorted.bam
bamToBed -bed12 -i reads.outfile.sorted.bam > reads.outfile.sorted.bed
#2: extract 5' (pTSS) and 3' (pTTS) mapping co-ordinates
awk '$6 ~ /^+$/ {print $0}' reads.outfile.sorted.bed | cut -f2 | sort -n | uniq -c | sed 's/^ *//' | sed 's/ /\t/g' | awk '{print "HSV1-st17",$2,$2,$1}' > forward.pTSS.txt
awk '$6 ~ /^-$/ {print $0}' reads.outfile.sorted.bed | cut -f3 | sort -n | uniq -c | sed 's/^ *//' | sed 's/ /\t/g' | awk '{print "HSV1-st17",$2,$2,$1}' > reverse.pTSS.txt
awk '$6 ~ /^+$/ {print $0}' reads.outfile.sorted.bed | cut -f3 | sort -n | uniq -c | sed 's/^ *//' | sed 's/ /\t/g' | awk '{print "HSV1-st17",$2,$2,$1}' > forward.pTTS.txt
awk '$6 ~ /^-$/ {print $0}' reads.outfile.sorted.bed | cut -f2 | sort -n | uniq -c | sed 's/^ *//' | sed 's/ /\t/g' | awk '{print "HSV1-st17",$2,$2,$1}' > reverse.pTTS.txt
---For visualization options, see section 'Visualization using Rstudio'
```

Identification of putative transcript fusions

-- Fusion transcripts are challenging to identify, particular for a virus such as HSV-1 where many genes are arranged in polycistronic units and resulting transcripts may encode multiple ORFs. For instance, in a polycistronic gene array arranged 1-2-3, all transcripts containing -1- will also contain -2- and -3- as all three genes will share the same poly(A) signal site. Thus, simply aligning reads against and HSV-1 transcriptome database is not sufficient to detect true chimeras.

```
#1:  First map the nanopore reads against a transcriptome database and extract reads that map to multiple ORFs.
minimap2 -ax splice -k14 -uf --secondary=no HSV1-st17.transcripts.fasta nanopore.fastq > aligned.sam
awk '$2==2048 {print $1}' aligned.sam | sort | uniq > chimeric.reads.list
#2: Generate a fasta file containing only full length chimeric pseudotranscripts
filterbyname.sh in=nanopore.fastq out=chimeric.reads.fasta names=chimeric.reads.list include=t overwrite=TRUE
#3: Identify all ORFs greater than 150 amino acids
```

```
ORFfinder -in chimeric.reads.fasta -outfmt 1 -ml  150 -n true -s 0 -strand plus
> chimeric.reads.orfs.fasta
# fix fasta file so each sequence is on one line only
awk '!/^>/ { printf "%s", $0; n = "\n" } /^>/ { print n $0; n = "" }END { printf "%s", n
}'  chimeric.reads.orfs.fasta > chimeric.reads.orfs.fixed.fasta

# Identify only full coding frames
awk 'NR%2{printf "%s ",$0;next;}1'  chimeric.reads.orfs.fixed.fasta | grep "TAA$" > TAA.txt
awk 'NR%2{printf "%s ",$0;next;}1'  chimeric.reads.orfs.fixed.fasta | grep "TGA$" > TGA.txt
awk 'NR%2{printf "%s ",$0;next;}1'  chimeric.reads.orfs.fixed.fasta | grep "TAG$" > TAG.txt
# Merge and output
cat TAA.txt TGA.txt TAG.txt | awk '{print $1,$2"\n"$3}' > complete.orfs.fasta
```

#4: Map predicted ORFs against the HSV-1 transcriptome and export IDs of all those mapping to multiple transcripts.

```
minimap2 -ax splice -k14 -uf --secondary=no HSV1-st17.transcripts.fasta complete.orfs.fasta >
aligned.sam
awk '$2==2048 {print $1}' aligned.sam > temp1.list
awk '$2==4 {print $1}' aligned.sam > temp2.list
cat temp1.list temp2.list | sort | uniq > temp.list
```

#5: Extract predicted ORF sequences with matching IDs, then map to the HSV-1 genome and print out all mappings with intronic regions. These represent a list of putative transcript fusions that can be taken forward for closer examination

```
filterbyname.sh in= complete.orfs.fasta out=candidates.fasta names=temp.list include=t
overwrite=TRUE
minimap2 -ax splice -k14 -uf --secondary=no / HSV1-
st17.genome.fasta candidates.fasta > fusions.sam
awk '$6 ~ /N/ {print $0}' fusions.sam
```

-- The final list should not be considered a gold standard by any means. At this stage we recommend visualising the original mapping of nanopore sequence reads to the genome to find and examine the splice sites used (Do they use canonical donors/acceptors? Are they supported by multiple reads?). subsequent experimental confirmation is, of course, absolutely essential.

Visualization using Rstudio

-- Most of the images in this manuscript are generated using Rstudio. Genome plots invariably make use of Gviz in concert with GenomicFeatures while scatter (violin) plots are made using ggplot2. Boundless examples of how to use these tools can be found all over the internet with specific pertinent examples available via the links below.

Genome visualizations

https://davetang.org/muse/2013/10/03/using-gviz/

Scatter (violin) plots

http://www.sthda.com/english/wiki/ggplot2-violin-plot-quick-start-guide-r-software-and-data-visualization

List of packages required for the example analyses

FLASh - https://ccb.jhu.edu/software/FLASH/

proovread - https://github.com/BioInf-Wuerzburg/proovread

bbtools - https://jgi.doe.gov/data-and-tools/bbtools/

SAMtools - http://samtools.sourceforge.net/

BEDtools - https://bedtools.readthedocs.io/en/latest/

ngsutils - https://github.com/ngsutils/ngsutils

Gviz - https://bioconductor.org/packages/release/bioc/html/Gviz.html

GenomicFeatures - https://bioconductor.org/packages/release/bioc/html/GenomicFeatures.html

ggplot2 - https://ggplot2.tidyverse.org/
